# Oral ketamine effects on dynamics of functional network connectivity in patients treated for chronic suicidality

**DOI:** 10.1007/s00406-024-01831-x

**Published:** 2024-05-21

**Authors:** Zack Y. Shan, Adem T. Can, Abdalla Z. Mohamed, Megan Dutton, Daniel F. Hermens, Vince D. Calhoun, Leanne M. Williams, Maxwell Bennett, Jim Lagopoulos

**Affiliations:** 1 Thompson Institute, University of the Sunshine Coast, 12 Innovation Parkway, Birtinya, QLD 4575 Australia; 2 Tri-institutional Center for Translational Research in Neuroimaging and Data Science (TReNDS), Georgia State University, Georgia Institute of Technology, Emory University, Atlanta, GA USA; 3 Department of Psychiatry and Behavioral Sciences, Stanford University, Stanford, CA USA; 4 University of Sydney, Sydney, NSW 2006 Australia; 5 Thompson Brain and Mind Healthcare, 55 Plaza Parade, Maroochydore, QLD 4558 Australia

**Keywords:** Ketamine, Dynamic functional connectivity, Chronic suicidality, Neural networks, fMRI, Brain

## Abstract

The underlying brain mechanisms of ketamine in treating chronic suicidality and the characteristics of patients who will benefit from ketamine treatment remain unclear. To address these gaps, we investigated temporal variations of brain functional synchronisation in patients with suicidality treated with ketamine in a 6-week open-label oral ketamine trial. The trial’s primary endpoint was the Beck Scale for Suicide Ideation (BSS). Patients who experienced greater than 50% improvement in BSS scores or had a BSS score less than 6 at the post-treatment and follow-up (10 weeks) visits were considered responders and persistent responders, respectively. The reoccurring and transient connectivity pattern (termed brain state) from 29 patients (45.6 years ± 14.5, 15 females) were investigated by dynamic functional connectivity analysis of resting-state functional MRI at the baseline, post-treatment, and follow-up. Post-treatment patients showed significantly more (FDR-Q = 0.03) transitions among whole brain states than at baseline. We also observed increased dwelling time (FDR-Q = 0.04) and frequency (FDR-Q = 0.04) of highly synchronised brain state at follow-up, which were significantly correlated with BSS scores (both FDR-Q = 0.008). At baseline, persistent responders had higher fractions (FDR-Q = 0.03, Cohen’s d = 1.39) of a cognitive control network state with high connectivities than non-responders. These findings suggested that ketamine enhanced brain changes among different synchronisation patterns and enabled high synchronisation patterns in the long term, providing a possible biological pathway for its suicide-prevention effects. Moreover, differences in cognitive control states at baseline may be used for precise ketamine treatment planning.

**Supplementary Information:**

The online version contains supplementary material available at 10.1007/s00406-024-01831-x.

## Introduction

Suicide remains a global problem and accounts for 1.4% of premature death [1]. Ketamine has been demonstrated as a rapid-acting and effective treatment for suicidality [2–4]. Two randomised [5, 6] and a double-blind, randomised, placebo-controlled trial have reported that ketamine has persistent benefits in patients with suicidal ideation. However, recent separate systematic reviews have reported inconsistent conclusions [4, 7]. One review suggests that ketamine failed to reduce suicidal ideation in patients with treatment-resistant depression [7], Another one reported that intravenous ketamine was superior to placebo or midazolam [4]. One of the potential reasons for the inconsistency in findings could be that ketamine is beneficial for some patients but not others. Thus, understanding the precise mechanisms behind ketamine’s mode of action is imperative, thus enabling the selection of patients who will benefit from ketamine treatment.

Ketamine’s actions have been studied at the molecular level in preclinical studies (for a review, see [8]) and at the organ level using neuroimaging. Preclinical studies report that ketamine selectively blocks N-methyl-D-aspartate receptors (NMDARs) on GABAergic inhibitory interneurons, leading to disinhibition of pyramidal neurons and enhanced glutamatergic firing [8]. While preclinical studies have shed light on potential pathways at the molecular level, functional MRI (fMRI) has bridged the gap between molecular pathways and symptom-related changes at the organ level. Kotoula et al. [9] systematically reviewed 12 resting state connectivity studies before September 2020 on the effects of subanaesthetic ketamine administration in healthy and depressed individuals. Preliminary conclusions were increased connectivity in reward and emotion processing areas in patients treated with ketamine [9]. More recently, Vasavada et al. [10] found that ketamine modulates functional connectivity between limbic regions and resting-state networks that were disrupted in depressed patients: increased connections between the right amygdala and the right central executive network, decreased connections between the left amygdala and the salience network. Mkrtchian et al. [11] observed that ketamine increased frontostriatal network (otherwise referred to as the reward network) connectivity in depressed patients towards levels observed in healthy controls. In summary, resting state fMRI connectivity analyses have provided valuable information concerning ketamine normalisation of reward and emotion processing networks. However, the process of achieving this normalisation is less clear, possibly due to the assumption that functional connectivity remains constant during the MRI scanning [12].

Dynamic functional connectivity and its network analogue, dynamic functional network connectivity (dFNC) analysis, investigates intrinsic brain networks in short time windows, providing a more temporally sensitive understanding of large-scale network activity dynamics [13]. dFNC analysis captures transient brain synchronisation patterns (termed brain states) that are averaged out in the static functional connectivity and their temporal variabilities. Each brain state represents a reoccurring and transient connectivity pattern among different regions underpinning brain functions [14]. The dFNC measures include the frequency of brain shifting among different brain states and the time and frequency of brain dwelling at each brain state.

To the best of our knowledge, this is the first study to investigate dFNC changes during ketamine treatment for chronic suicidality. Based on preclinical findings of ketamine’s effects on the glutamatergic system, we hypothesised that patients treated with ketamine for suicidality would have more temporal variations in brain states at post-treatment and follow-up than those at baseline. In addition, we were interested in exploring if there are differences in the dFNC measures at the baseline between those from the responders and non-responders. Here, we investigated dFNC measures of the whole brain and individual intrinsic networks, as each network may exhibit unique brain states and characteristics.

## Methods

### Ethics approval

Ethics approval was obtained through Bellberry Limited (2017-12-982) and ratified by the University of the Sunshine Coast Human Research Ethics Committee (A181101). A written consent form was obtained and signed by each participant. This study was registered with the Australian Clinical Trials Registry (ACTRN12618001412224).

### Patients and study design

This retrospective study analysed all available MRI and clinical data from a prospective cohort, participating in an open-label clinical trial of ketamine treatment in adults with chronic suicidality. The demographics of the participants have been reported previously [3]. In brief, patients presenting with chronic suicidality were recruited. Chronic suicidality was defined as experiencing varying-intensity suicidal ideation continuously or intermittently over months to years, with an ongoing risk of considering a future attempt, as determined by the study psychiatrist (AC) and further reinforced by a Beck Scale for Suicidal Ideation (BSS) score greater than 6. Recruitment was agnostic to DSM diagnosis, and as such, participants with a broad range of diagnoses within the community were recruited and included borderline personality disorder, generalised anxiety disorder, major depressive disorder, obsessive-compulsive disorder, panic disorder, post-natal depression, post-traumatic stress disorder, and substance use disorder (in remission). Participants adhered to their prescribed medication regimen, which included selective serotonin reuptake inhibitors, serotonin and norepinephrine reuptake inhibitors, and mood stabilisers throughout the treatment and follow-up phases. Our previous publication details the specific psychotropic medications taken by each participant [3]. Medication status did not impact the eligibility of participants to enter the study. The treatment lasted 6 weeks, at one dose per week with an initial dose of 0.5 mg/kg and the maximal last dose of 3.0 mg/kg. The dosage was up-titrated by 0.2 to 0.5 mg/kg or down-titrated by 0.2 to 0.7 mg/kg each week, depending on patient tolerance and response, as determined by the study psychiatrist (AC) [3]. MRI and clinical assessments were performed at baseline (prior to first treatment), post-treatment (1–7 days after the last dose) and follow-up (28–32 days after the last dose).

### MRI

MRI scans were performed on a 3-Tesla Siemens Skyra MRI (Germany, Erlangen) with a 64-channel head and neck receive coil. The T1-weighted structural and resting-state fMRI were used in this study. The structural images were acquired using a 3D Magnetization-Prepared Rapid-Acquisition Gradient Echo sequence with a time of repetition (TR) = 2.2 s, echo time = 1.76 ms, a field of view (FOV) = 240 × 240 mm^2^, matrix resolution = 256 × 256, spatial resolution = isotropic 0.9 mm^3^, and 208 slices. Resting-state fMRI was acquired in the eyes closed condition using an echo-planar imaging multiband sequence with TR = 1.4 s, a FOV = 240 × 240 mm^2^, matrix resolution = 80 × 80, spatial resolution = isotropic 3 mm^3^, 56 slices, multi-slice factor = 4, acceleration factor = 2, 404 volumes, and the total collection time of 565.6 s.

### Clinical assessments

This study used BSS and Montgometry-Asberg Depression Rating Scale (MADRS) scores as primary and secondary outcome measures, respectively. The MADRS was chosen as the secondary outcome measure since it was predicted that all participants would exhibit depressive symptoms [3]. The BSS was assessed at baseline, post-treatment, and follow-up. The MADRS was assessed at the baseline and follow-up time-points. The BSS is a 21-item clinical rating instrument designed to quantify and evaluate suicidal intention by addressing suicidal thoughts, their characteristics and feelings, and plans regarding suicide, with a total score ranging from 0 (no risk) to 42 (highest suicidality) [15]. The MADRS is a ten-item diagnostic questionnaire measuring the severity of depressive symptoms, with a total score ranging from 0 (normal) to 60 (most severe depression) [16]. Responders and persistent responders were defined as patients with greater than 50% improvement in BSS scores or a BSS score less than 6 at the post-treatment and follow-up visit, respectively.

### MRI pre-processing, image quality and motion control

The standard pre-processing implemented in SPM12 (http://www.fil.ion.ucl.ac.uk/spm/) was used, which included (i) the 2-pass motion correction, (ii) co-registration of fMRI volumes to the structural images, (iii) structural images were normalised to the standard space, (iv) the normalisation matrices found in (iii) were applied to co-register fMRI data to normalise them into the standard space; (v) a 6 mm Gaussian kernel was used to smooth fMRI volumes.

The selection of dFNC analysis parameters was related to the TR. We excluded 2 datasets because the data were accidentally acquired with TR = 1.6 s. Another 4 datasets were removed because of excessive motion: either (i) a data set that had > 5% volumes with head displacement > 3 mm, (ii) a data set that had > 15% volumes with frame-to-frame displacement > 0.4 mm; or (iii) a data set that correlated its mask with the averaged mask less than 0.9 (an outlier dataset not in the standard space).

### Intrinsic network identification

**Fig. 1:**
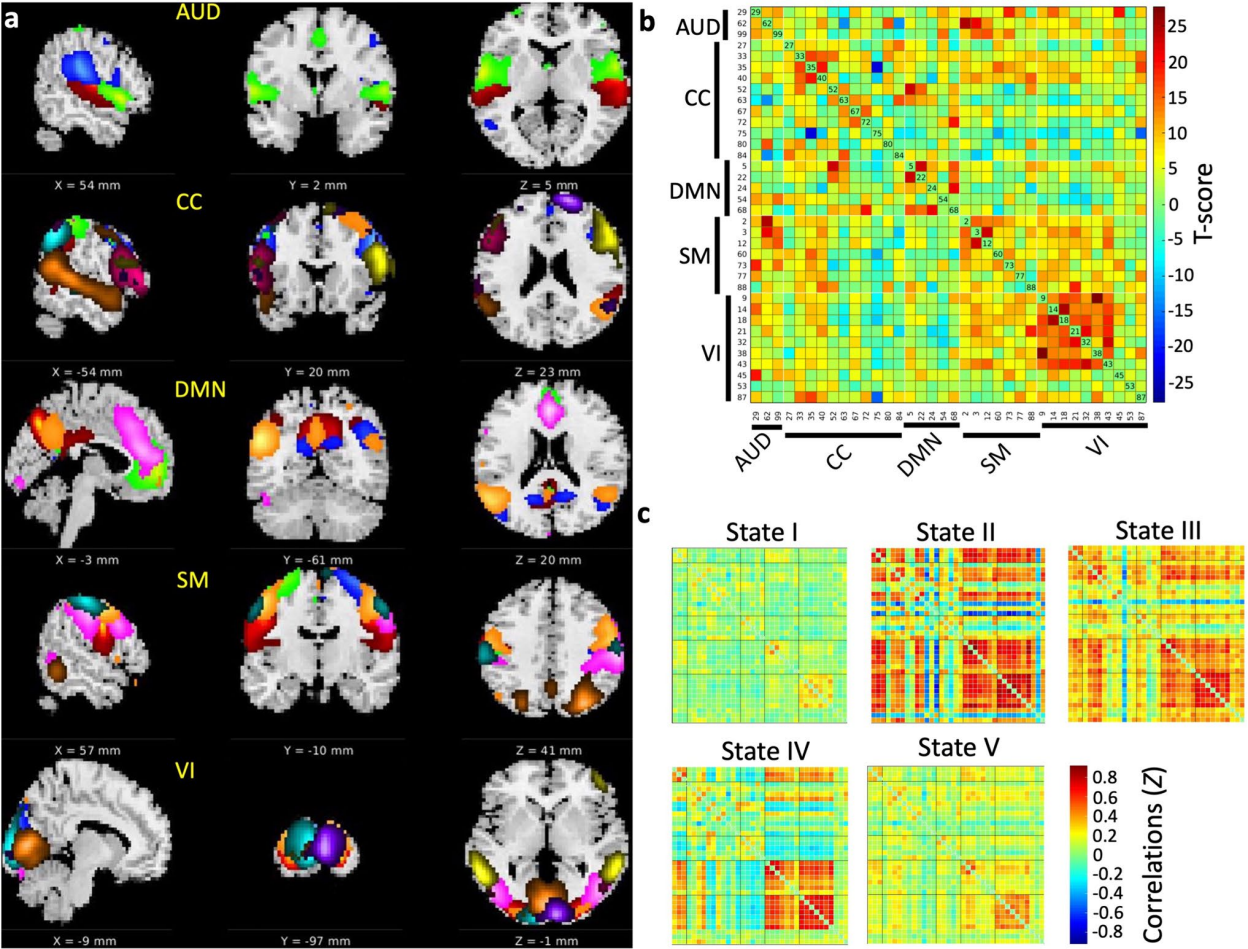
Intrinsic networks (36 independent components, ICs) and 5 brain states identified by group IC and dynamic functional network (DFNC) analyses. (**a**) Spatial maps of 36 ICs were labelled using GIFT software (GroupICAT 4.0c) and overlaid on an averaged structural image, *AUD *auditory network, *CC *cognitive control network, *DMN *default mode network, *SM *sensorimotor network, *VI *visual network. (**b**) The averaged static functional connectivity matrix was calculated as correlations between each IC pair and converted into T-scores. The ICs were sorted according to labelled networks. (**c**) The matrices represented correlation *z*-scores of 5 transient and reoccurring brain connectivity patterns (termed brain states) by the K-means clustering. The brain state II had the highest, while state I had the lowest correlations. Correlations in brain states III, IV, and V were between those in states I and II.

We used the GIFT (GroupICAT 4.0c, https://trendscenter.org/software/gift/) [17] group spatial independent component (IC) analysis to identify intrinsic networks from pre-processed resting-state fMRI. (i) A principal component analysis was used to reduce each data set to 120 components. (ii) The Infomax IC analysis from the ICASSO [18] was repeated 20 times to estimate 100 ICs and ensure the replicability of ICs. (iii) The aggregate components of IC analysis [19] were used to back-reconstruct the IC spatial maps. (iv) Two experienced scientists (AZM and ZYS) manually identified intrinsic network following previously described criteria [12]: (a) the peak z-scores of IC spatial maps located in grey matter area, (b) IC spatial maps not likely originated from motion (edging maps) or located in ventricular areas, (c) ICs having time courses dominated by with low frequency (< 0.1 Hz) power, (d) ICs having time courses with a high dynamic range difference between the minimum and maximum power frequencies. We identified 36 ICs as intrinsic networks. The 36 ICs were labelled automatically (component labelling in GIFT) based on spatial correlations with the neuromarker 53 template (Fig. 1a, b).

### dFNC analysis and its measures

We used the dFNC toolbox implemented in the GIFT and established procedures [12, 13, 20] to conduct the dFNC analysis of the whole brain and individual networks. In brief, (i) time courses of ICs were detrended to remove linear, quadratic, and cubic trends and filtered with a cut-off frequency of 0.15 Hz. (ii) Each time course was divided using a sliding window method with a step between windows of 1 TR into segments with a length of 30 TR (42 s). The tampered window was created by convolving a 30 TR (42 s) width rectangle with a Gaussian (σ = 3 TR) filter. (iii) Sparse inverse covariance matrices were computed using the L1 penalty to estimate time course segment connectivities with 100 repetitions. (iv) City distances were calculated to estimate the similarities between any pair of time course segments’ connectivity matrices. K-means clustering was used to group similar connectivity matrices as a cluster (termed brain state). The optimal number of brain states was determined using the elbow criterion.

The dFNC properties for the whole brain and each network include the number of transitions (*N*_*T*_), the mean dwell time (*D*) of each brain state, and the fraction (*f*) of each brain state. The number of transitions is the number of times a brain state changes into another. The mean dwell time is calculated by the number of full sliding windows (42s) of a brain state divided by the number of transitions entering this state. The fraction of each brain state is calculated by the total length of a brain state divided by the total length of resting-state fMRI data.

### Statistical analysis of dFNC properties and clinical data

The brain and network dFNC properties and relationship with psychological data were assessed using SPSS® Version 28 (SPSS Inc., Chicago, Illinois, USA). The Shapiro-Wilk test was used to test for normality, and equivalent non-parametric tests were performed for variables that were not normally distributed. Independent samples t-tests with two-tails and no equivalent variance assumption were used to compare the dFNC differences between responders vs. non-responders and persistent responders vs. non-responders at baseline. Paired-samples-tests with two-tails were used to compare the dFNC changes and symptom measures between baseline vs. post-treatment and baseline vs. follow-up. Spearman correlations with two-tails were used to examine monotonic relationships between dFNC and behaviour measures. The false discovery rate (FDR-Q) was calculated using the Benjamini-Hochberg method for multiple comparison correction with a significance threshold of FDR-Q < 0.05. The missing data were excluded using SPSS analysis-by-analysis option, which handles missing data through pairwise deletion.

## Results

### Patients and symptom score changes

Thirty patients completed the ketamine treatment and follow-up visit; however, one of them did not undergo MRI scans. During the study period, no significant adverse events were documented. All patients who received ketamine exhibited a favourable tolerability profile, evidenced by the absence of participant withdrawals attributed to ketamine-related side effects. Among the observed side effects, the most reported included reduced energy levels and fatigue, followed by instances of anxiety, impaired concentration, restlessness, generalised malaise, dry mouth, dizziness, and tremors.

**Fig. 2:**
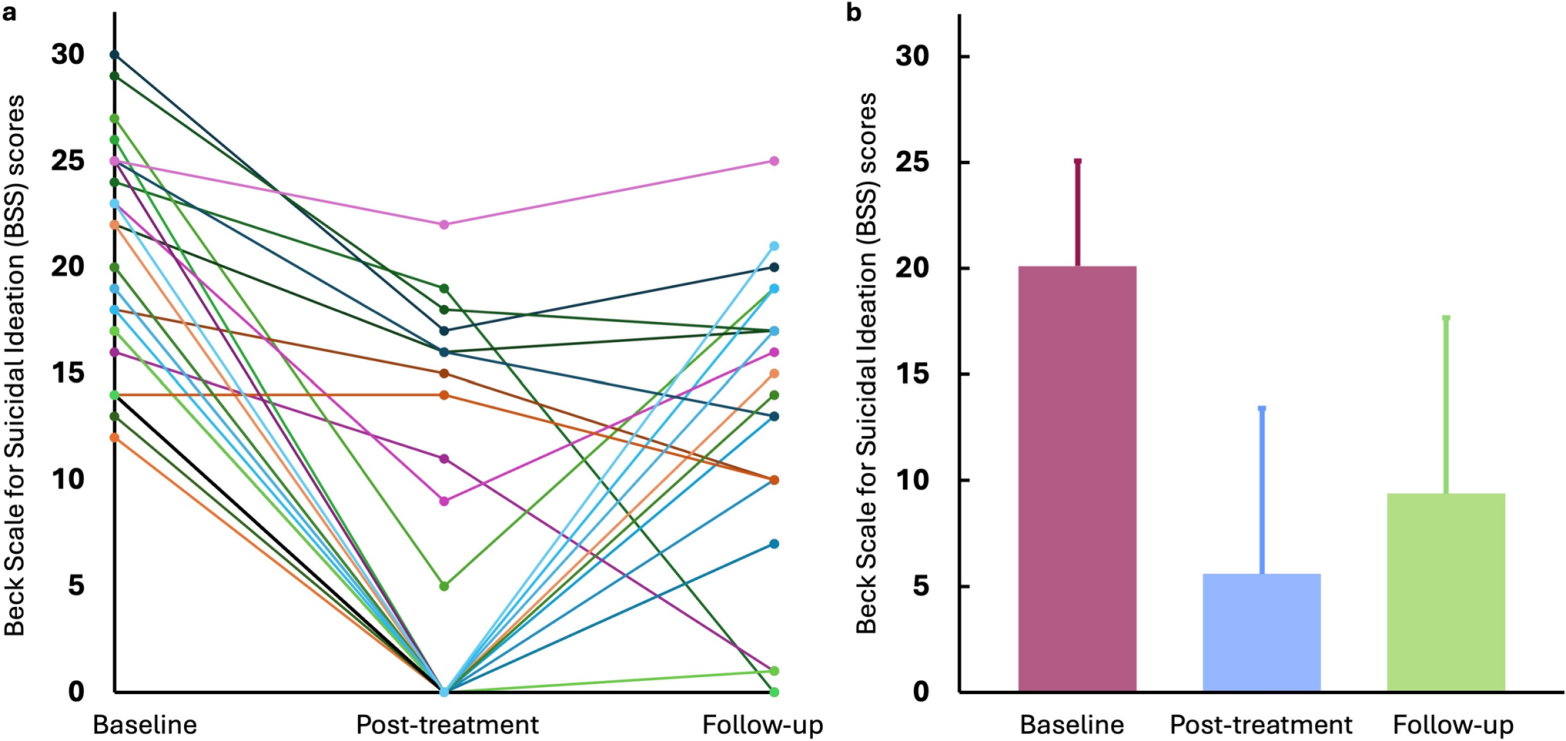
Individual and mean Beck Scale for Suicidal Ideation (BSS) scores across three-time points in the final sample. (**a**) A scatter plot illustrated the BSS scores of each patient, connected with lines to depict individual changes from the baseline to post-treatment and follow-up. (**b**) A bar graph showed the mean BSS scores of the final sample at the baseline, post-treatment and follow-up with error bars representing positive standard deviations. Colours were used solely for visual distinction

The current study retrieved all available MRI data from 29 participants (45.6 ± 14.46 years old), including 15 females and 14 males. The BSS scores at the post-treatment (5.59 ± 7.82) and the follow-up (9.38 ± 8.28) were significantly lower (both *P* < 0.001) than those at the baseline (20.1 ± 4.97). The individual and mean BSS scores at three-time points are summarised in Fig. 2. The MADRS scores at follow-up (16.21 ± 11.88) were significantly lower (*P* < 0.001) than at baseline (38.55 ± 7.81). Twenty and 15 patients were responders (50% improvement in BSS score or BSS score less than 6) and persistent responders, respectively. Six MRI datasets were excluded because of image qualities and inconsistent TR parameters.

### Whole brain dFNC states, changes, and their relationships with psychological measures

Five whole brain dFNC states were identified (Fig. 1c). States I and II had the lowest and highest correlations among the 36 ICs, respectively, while states III, IV, and V had connectivity strength in between.

**Fig. 3:**
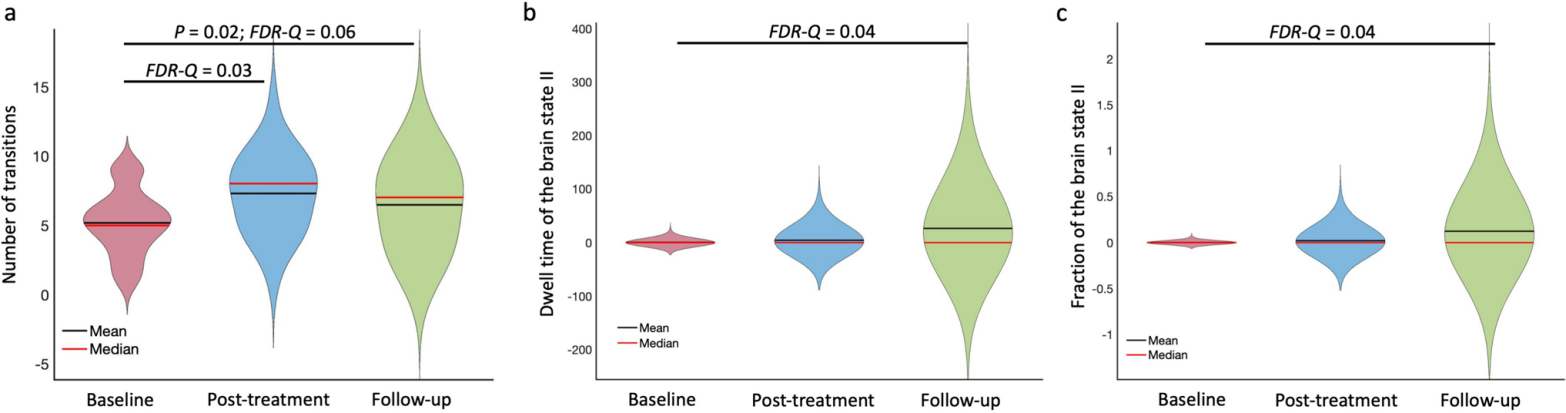
Differences of the whole brain dynamic functional connectivity measures among the baseline, post-treatment, and follow-up. (**a**) Compared with the baseline, the brain states transited more frequently (*P* = 0.003, *FDR-Q* = 0.03) post-treatment and more frequently (*P* = 0.02, *FDR-Q* = 0.06) at the follow-up. (**b**) The mean dwell time, measured as numbers of sliding windows (42s), of the brain state II at the follow-up was significantly greater (*P* = 0.005, *FDR-Q* = 0.04) than those at the baseline. (**c**) The fraction of the brain state II at the follow-up was significantly higher (*P* = 0.008, *FDR-Q* = 0.04) than at the baseline

The number of transitions among 5 brain states at post-treatment (*N*_*T*_ = 7.38 ± 2.97) was significantly higher (*P* = 0.003, *FDR-Q* = 0.03) than those at baseline (*N*_*T*_ = 5.19 ± 2.35) (Fig. 3a). However, no significant differences (FDR-Q > 0.05) were observed in the mean dwell time or the fraction of each brain state between baseline and post-treatment.

**Fig. 4:**
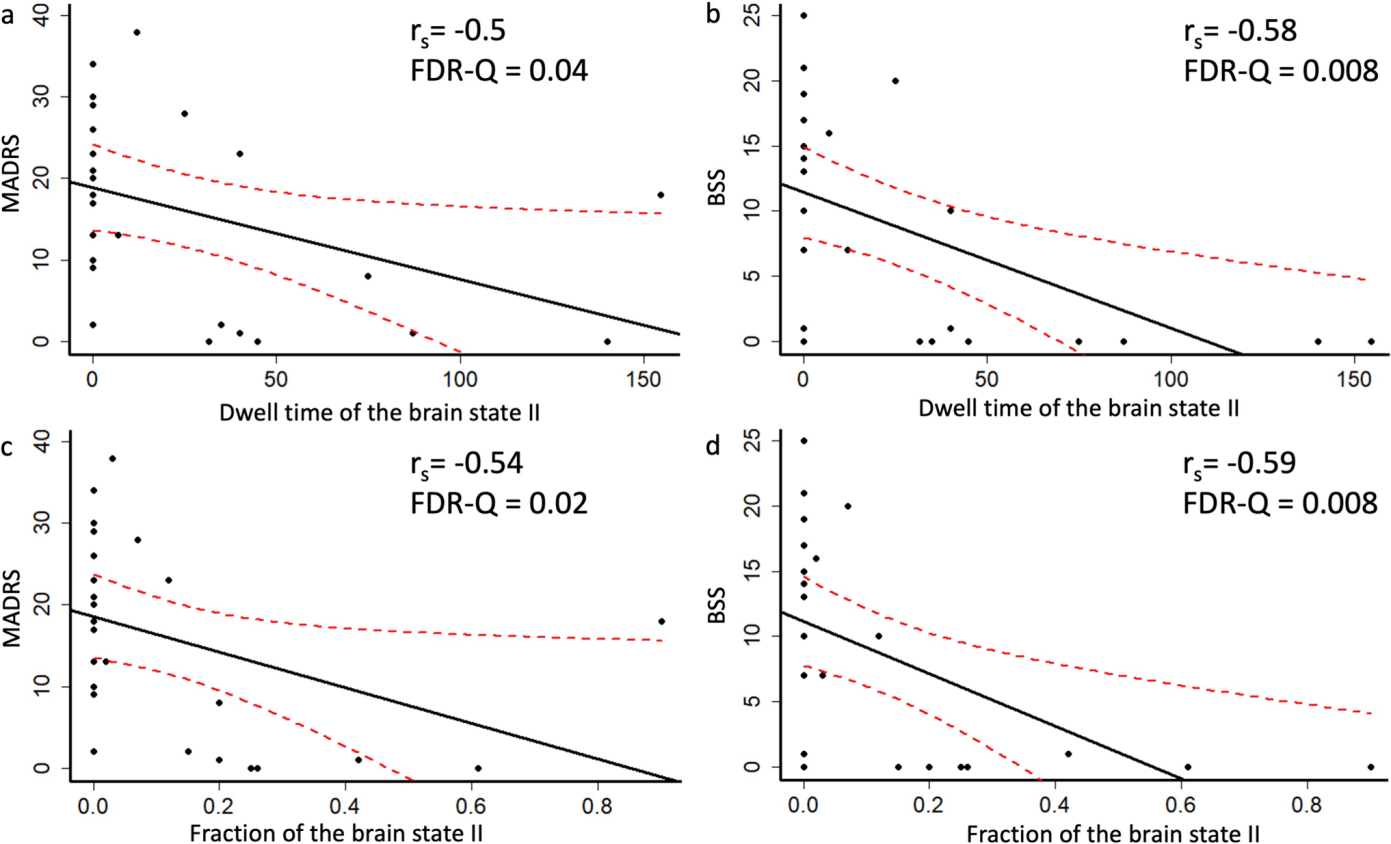
Associations of the whole brain dynamic functional connectivity with psychological measures at the follow-up. A linear regression (black line) was fitted, and red dashes indicated the 95% confidence interval. The Montgometry-Asberg Depression Rating Scale (MADRS) and Beck Scale for Suicidal Ideation (BSS) measured symptom severity with higher scores for worse symptoms. Spearman (*r*_*s*_) correlations were tested. The dwell times of brain state II were negatively and significantly correlated with MADRS (**a**) and BSS (**b**) scores. The fractions of brain state II were negatively and significantly correlated with MADRS (**c**) and BSS (**d**)

The number of transitions at the follow-up time-point (*N*_*T*_ = 6.92 ± 3.05) was higher (*P* = 0.02, *FDR-Q* = 0.06) than at the baseline (Fig. 3a). The mean dwell time (*D*_*II*_*=* 28.84 ± 44.21 measured in the number of 42s sliding window) and the fraction (*f*_*II*_ = 0.13 ± 0.22) of the brain state II at the follow-up was significantly greater (*D*_*II*_: *P* = 0.005, *FDR-Q* = 0.04; *f*_*II*_: *P* = 0.008, *FDR-Q* = 0.04) than at the baseline (*D*_*II*_*=* 0.58 ± 2.86; *f*_*II*_ = 0.002 ± 0.008) (Fig. 3b, c). Moreover, the mean dwelling time and the fraction of brain state II at the follow-up was significantly and negatively correlated with the MADRS (*D*_*II*_: *P* = 0.009, *FDR-Q* = 0.04, *r*_*s*_ = − 0.5; *f*_*II*_: *P* = 0.004, *FDR-Q* = 0.02, *r*_*s*_ = − 0.54) (Fig. 4a, c) and BSS scores (*D*_*II*_: *P* = 0.002, *FDR-Q* = 0.008, *r*_*s*_ = − 0.58; *f*_*II*_: *P* = 0.002, *FDR-Q* = 0.008, *r*_*s*_ = − 0.59) (Fig. 4b, d). Finally, we did not observe any significant differences (FDR-Q > 0.05) in the mean dwelling time and fraction in the brain states I, III, or IV between baseline and follow-up.

### Network dFNC states, changes, and their relationships with clinical measures

For each network, four brain states were identified (Supplementary Fig. 1).

**Fig. 5:**
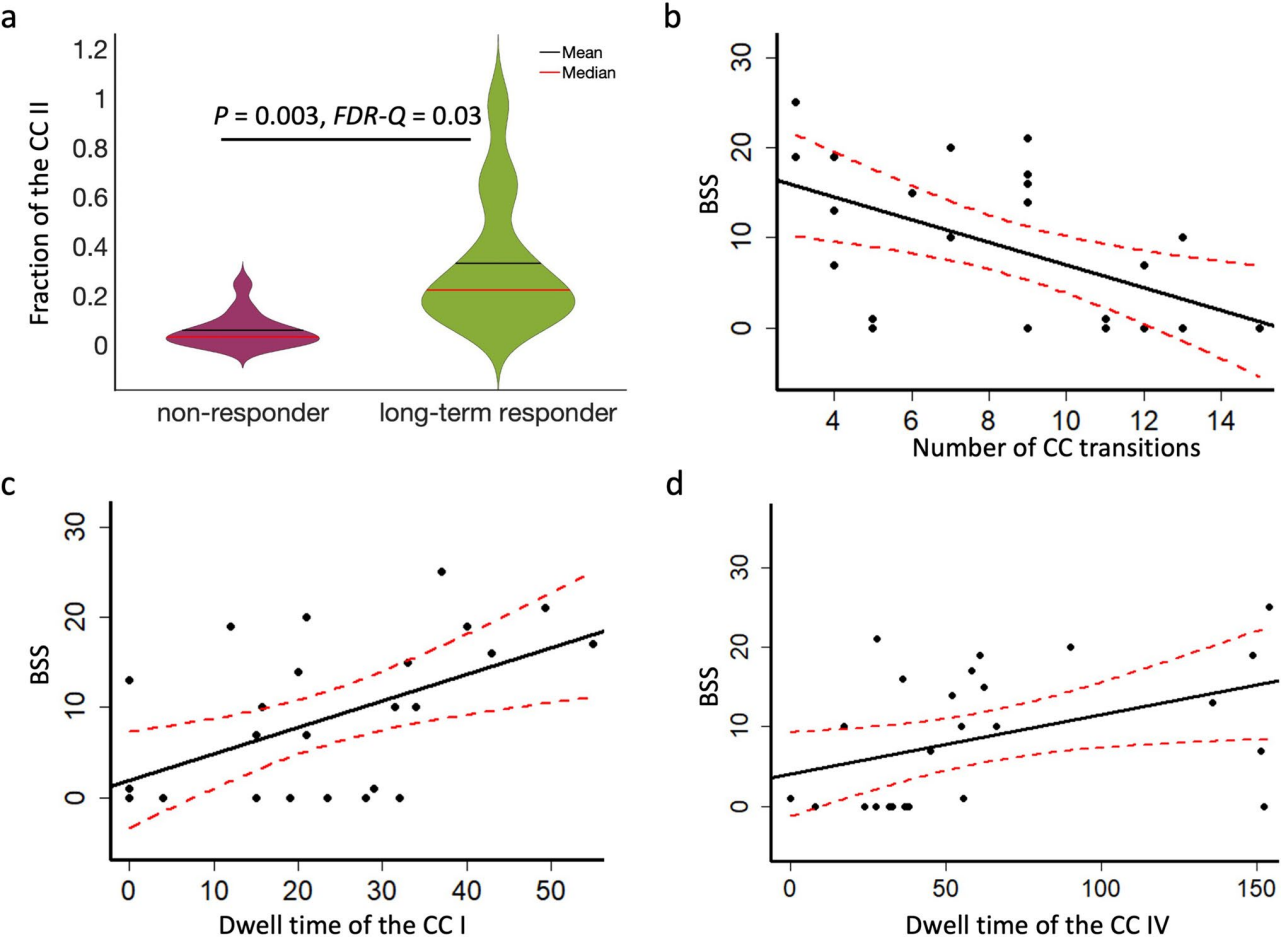
The cognitive network (CC) dynamic functional connectivity measures and their relationships with psychological scores. (**a**) The persistent responders have significantly higher fractions of CC II (*P* = 0.003, *FDR-Q* = 0.03, Cohen’s *d* = 1.39) at the baseline than non-responders. (**b**) The numbers of transitioning among CC states at the follow-up were significantly and negatively correlated (*P* = 0.003, *FDR-Q* = 0.03, *r*_*s*_ = − 0.55) with the Beck Scale for Suicidal Ideation (BSS) scores. (**c**) The dwell times, measured as numbers of sliding windows (42 s), of CC I were significantly and positively correlated (*P* = 0.006, *FDR-Q* = 0.03, *r*_*s*_ = 0.52) with BSS scores at the follow-up. (**d**) The dwell times of CC IV were significantly and positively correlated (*P* = 0.01, *FDR-Q* = 0.05, *r*_*s*_ = 0.47) with BSS scores at the follow-up

Among the four brain states of the cognitive network (CC), states III and IV have the highest and lowest connectivities, respectively. In contrast, states I and II have connectivity strengths in between (Supplementary Fig. 1). The fraction of CC II at baseline of the persistent responders (*f*_*II*_ = 0.33 ± 0.27) was significantly higher (*P* = 0.003, *FDR-Q* = 0.03, Cohen’s *d* = 1.39) than those of the non-responder (*f*_*II*_ = 0.06 ± 0.07) (Fig. 5a).

The dwell times of CC II at post-treatment (*D*_*II*_*=* 18.76 ± 20.72) was reduced compared with those at the baseline (*D*_*II*_*=* 42.84 ± 68.18), but this effect was not significant (*P* = 0.05, *FDR-Q* > 0.05). The fraction of CC in state II at post-treatment (*f*_*II*_*=* 0.13 ± 0.18) was reduced compared with those at baseline (*f*_*II*_*=* 0.21 ± 0.24), but not significantly so (*P* = 0.03, *FDR-Q* > 0.05).

The numbers of transitions among CC states and dwelling times in CC I at post-treatment was correlated with the BSS scores but this did not reach significance (*N*_*T*_ : *P* = 0.05, *FDR-Q* > 0.05, *r*_*s*_ = − 0.38; *D*_*I*_ : *P* = 0.02, *FDR-Q* > 0.05, *r*_*s*_ = 0.44). At the follow-up, the numbers of transitioning among CC states and dwelling times in CC I and CC IV was significantly correlated with the BSS score (*N*_*T*_ : *P* = 0.003, *FDR-Q* = 0.03, *r*_*s*_ = − 0.55; *D*_*I*_ : *P* = 0.006, *FDR-Q* = 0.03, *r*_*s*_ = 0.52; *D*_*IV*_ : *P* = 0.02, *FDR-Q* = 0.05, *r*_*s*_ = 0.47) (Fig. 5b–d).

**Fig. 6:**
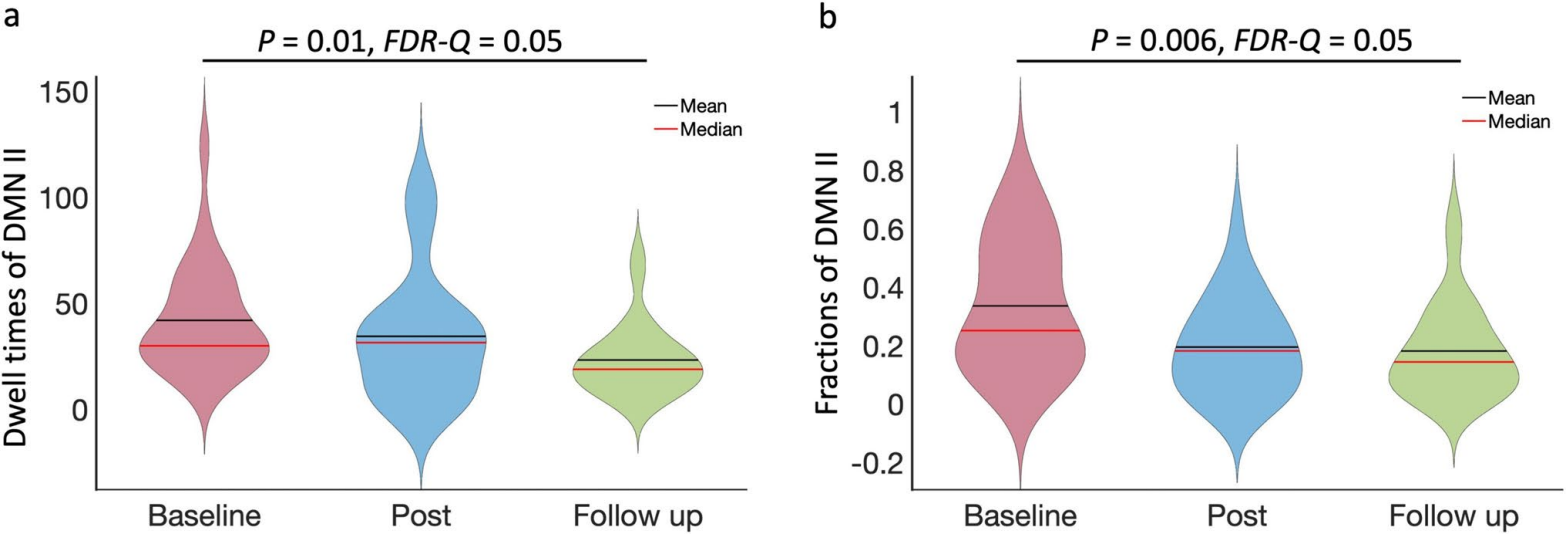
The default mode network (DMN) dynamic functional connectivity changes between the baseline, the post-treatment, and the follow-up. (**a**) The dwell times of DMN II, measured as numbers of sliding windows (42 s), at the follow-up were significantly lower (*P* = 0.01, *FDR-Q* = 0.05) than those at the baseline. (**b**) Compared with the baseline, fractions of DMN II at the follow-up were significantly lower (*P* = 0.006, *FDR-Q* = 0.05).

Among the four brain states of the default mode network (DMN), states IV and III have the highest and lowest connectivity, respectively, whilst states I and II have connectivity strengths in the middle (Supplementary Fig. 1). The fraction of DMN in state II at post-treatment (*f*_*II*_*=* 0.2 ± 0.16) was reduced compared baseline (*f*_*II*_*=* 0.33 ± 0.22), but not significantly so (*P* = 0.008, *FDR-Q* > 0.05). At follow-up, dwell times (*D*_*II*_*=* 23.88 ± 17.52) and the fractions (*f*_*II*_*=* 0.19 ± 0.17) of DMN in state II were reduced (*D*_*II*_: *P* = 0.01, *FDR-Q* = 0.05; *f*_*II*_: *P* = 0.006, *FDR-Q* = 0.05) compared with those at the baseline (*D*_*II*_*=* 40.92 ± 26.45, *f*_*II*_*=* 0.33 ± 0.22) (Fig. 6).

Although none of the following relationships were significant, the fractions of DMN IV were positively correlated (*P* = 0.02, *FDR-Q* > 0.05, *r*_*s*_ = 0.46) with BSS scores at the baseline; dwelling time of DMN I were negatively correlated (*P* = 0.02, *FDR-Q* > 0.05, *r*_*s*_ = − 0.46) with MADRS scores at the follow-up; fractions of DMN I were negatively correlated (*P* = 0.04, *FDR-Q* > 0.05, *r*_*s*_ = − 0.41) with BSS scores at the follow-up.

No significant differences (FDR-Q > 0.05) were observed in the dFNC measures of the auditory network, the sensorimotor network, and the visual network across the three time-points, nor were any significant correlations observed between the dFNC measures and any behaviour measure.

## Discussion

We found that the whole brain shifted more frequently and dwelled longer and more frequently on a brain state with the highest connectivity (state II) after treatment and at follow-up. Importantly, the dwelling times and fractions of brain state II were correlated with symptom improvement. We also found that persistent responders had high fractions of the CC state with high connectivity, which suggested the dFNC features could be potentially used to select patients who will respond to the ketamine. The strengths of this study were: (i) dFNC measures provide a more granular understanding of complex brain connections, including temporal changes and connectivity flexibilities, and (ii) the follow-up timepoint enabled the exploration of brain features in patients who benefit from ketamine treatment in the long term (10 weeks).

There was no direct comparison of consistency between our results with previous studies because this is the first study on dFNC changes associated with ketamine treatment. Meanwhile, the dFNC changes observed in our study at the organ level were consistent with previous mechanisms at the molecular level. Ketamine is an antagonist of the NMDAR and preferentially binds NMDARs expressed on GABAergic interneurons [21, 22]. Therefore, a subanesthetic dose of ketamine significantly increases extracellular glutamate levels [22] and glutamate cycling [23]. Glutamate is the primary excitatory neurotransmitter, and increased excitatory neurotransmitter levels may result in additional synaptic activations, manifesting as more frequent BOLD signal synchronisation changes. Brain state captures the dynamic changes in fluctuating functional connectivity patterns over time, reflecting the varying levels of coordination and communication between brain regions at different moments. Increased dwelling time and fractions at the brain state with high connectivities support this mechanism. The increased brain flexibility (numbers of transitions among brain states) provides a possible underlying process of brain function normalisation observed in static resting fMRI studies [9–11]. We postulated that brain synchronisations in patients with chronic suicidality were stationed in an abnormal pattern and that frequent brain state transitions aided in shifting out from this abnormal pattern.

While the efficacy of ketamine in treating suicidality is promising, potential risks of ketamine abuse remain. Ketamine can be additive [24, 25] and, when abused, can result in psychiatric, psychotomimetic, cardiovascular, and neurological side effects [24–26]. Thus, it is crucial to understand from a mechanistic perspective those patients who stand to benefit from ketamine treatment. The significantly greater fractions in CC II (second highest connectivity CC state, Supplementary Fig. 1 and Fig. 5a) in persistent responders than those in non-responders suggested that dFNC features of CC could be used to select patients. It has been well documented that the CC network plays a vital role in depression [27, 28]. A recent study has shown that whole brain dFNC measures can be used to identify suicidality in patients with major depressive disorders [29]. Our results suggest that ketamine has benefited patients with a certain level of CC reserve (persistent responders) but not patients with significant loss of CC functions (non-responders). However, our data could not determine whether the predictor was specific to ketamine or if patients with a certain level of CC reserve would also benefit from other treatments. Indeed, a recent study has reported that whole-brain dFNC matrices enhanced the prediction accuracy of antidepressant efficacy [30]. Furthermore, cognitive reserve (visual memory and low levels of mental processing speed) was predictive of a good response to bupropion for major depressive disorders [31]. It is also worth noting that body mass indices, Social and Occupational Functioning Assessment Scales, MADRS scores, number of suicide attempts, employment status, and age also contribute to differentiating responders and non-responders [32].

The change in dFNC measures of individual networks explained the symptom improvements. Rumination is a well-known feature associated with active suicidal ideation in depression [33, 34]. One of the main functions of the DMN is self-referential operations [35]. Not surprisingly, abnormal DMN connectivity is tightly associated with suicidal ideation [36]. Our study identified 4 DMN states, and DMN II and IV had high connectivities while those in DMN I and III had low ones (Supplementary Fig. 1). Thus, significantly reduced dwell times and fractions of DMN II at the follow-up time-point might provide a neural substrate of symptom improvement (Fig. 6). The correlations between dwelling times and fractions of brain states and BSS and MADRS scores further supported this notion, although not significant after correction for multiple comparisons. We believe that the observed lack of significance in the correlations came down to the small sample size in our pilot study. Similar findings were observed on the CC state. CC II and III had high connectivities, while CC I and IV had low synchronisations (Supplementary Fig. 1). The transition numbers among different CC states indicates CC flexibility and provides another biological substrate of symptom improvement. Higher CC flexibility and synchronisation were associated with lower BSS scores (Fig. 5).

There are several limitations associated with this study that require discussion. Firstly, our study was based on an open-label design and did not incorporate a healthy control group. Ideally, a blinded and randomised study would have provided a greater ability to generalise our findings more broadly and also link brain states to ketamine efficacy. This not being the case, the results of our study could have been influenced by factors other than the treatment itself, such as expectancy, practice, or exposure effects. Also, the small sample size in our study was a limitation as it may have contributed to false negative and positive results. Indeed, we observed that multiple changes and associations were not statistically significant after multiple comparison corrections. The CC defined by the GIFT may include multiple networks with distinct roles in suicidality. However, due to the small sample size, our study followed a standard dFNC analysis process. Future studies with larger sample sizes are required to investigate networks at fine spatial scales. Next, our study investigated dFNC changes in patients with chronic suicidality treated with ketamine. Thus, the results may not apply directly to acute suicidality. Further, our study investigated the feasibility and safety of oral ketamine treatment in adults with ages ranging from 22.19 to 71.8 years old and hence the finding may not be directly applicable to children and adolescents. Post-treatment MRI scans were performed 1–3 days after the 6-week treatment without measuring ketamine distributions, which might introduce additional variances.

In conclusion, our results suggest that ketamine enhances the flexibility in brain connections and brain states with high synchronisations and that these changes are associated with concomitant symptom improvement. Finally, the CC network state differed at baseline between persistent responders and non-responders, this may suggest that the CC could be used for precise ketamine treatment planning in chronic suicidality.

## Electronic supplementary material

Below is the link to the electronic supplementary material.

Supplementary file1 (DOCX 490 KB)
