# Supplementary material for: Oral ketamine effects on dynamics of functional network connectivity in patients treated for chronic suicidality
Source: Eur Arch Psychiatry Clin Neurosci. 2024 May 21;275(5):1347–57. doi: 10.1007/s00406-024-01831-x (PMC12271241; doi:10.1007/s00406-024-01831-x)
Supplement: Supplementary file 1 — Supplementary file1 (DOCX 490 KB) [file 406_2024_1831_MOESM1_ESM.docx]

**Supplementary Figures**

Shan ZY et al. *Oral ketamine effects on dynamics of functional network connectivity in patients treated for chronic suicidality*


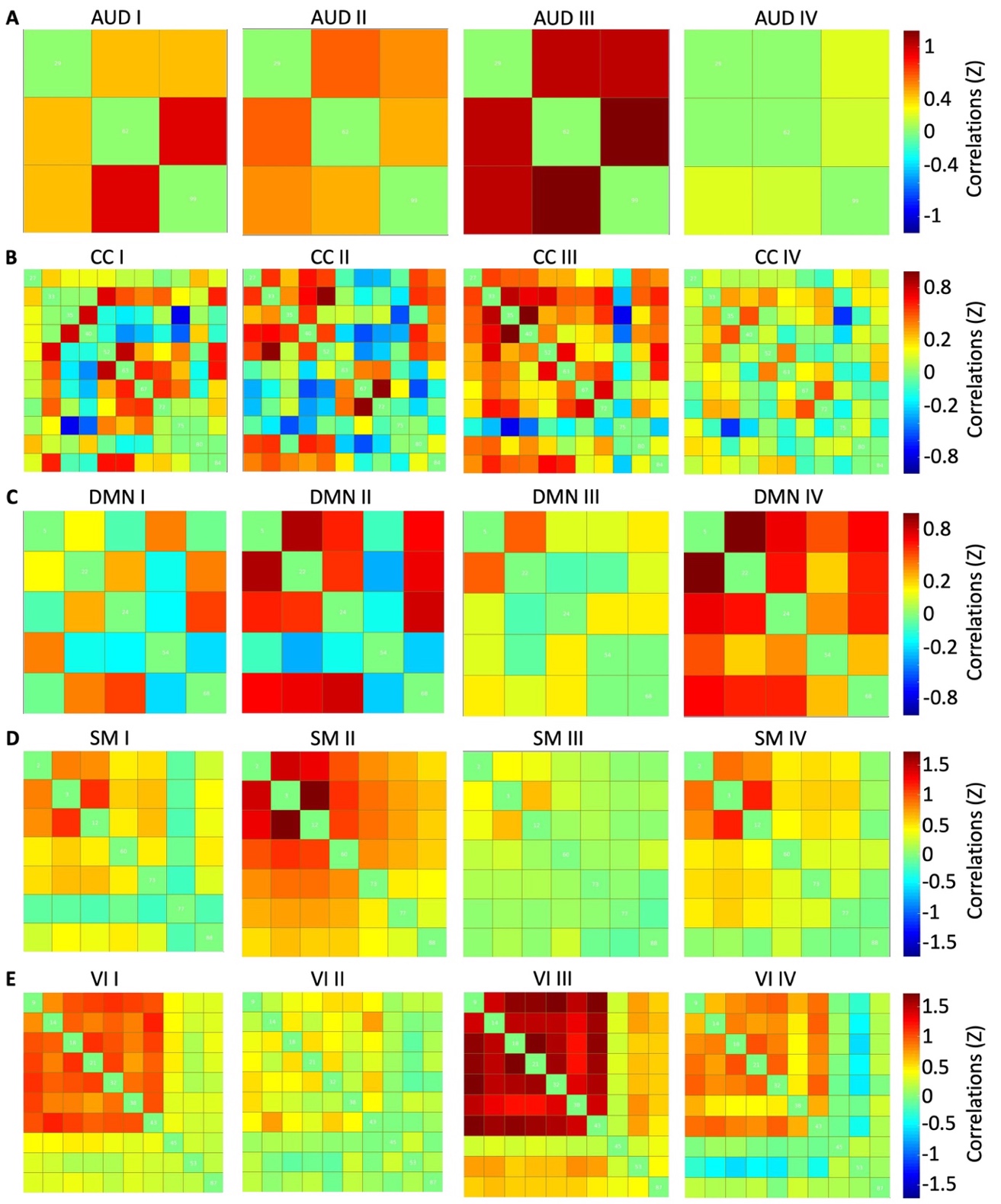


**Supplementary Figure 1 Network brain state centroids identified by the K-means clustering.** Four brain states were identified within each brain network. The correlation z-scores of each brain state centroid were encoded by the colour bars and the same colour bar was used for each network. (**A**) the auditory network (AUD), (**B**) the cognitive network (CC), (**C**) the default mode network (DMN), (**D**) the sensorimotor network (SM), and (**E**) the visual network (VI).
